# Therapeutic Effect of Tranilast on Lung Tumors Suspected of Being Staple Line Granulomas: Report of Two Cases

**DOI:** 10.70352/scrj.cr.25-0234

**Published:** 2025-07-01

**Authors:** Ayato Ura, Yoshifumi Shimada, Takahiro Homma, Keitaro Tanabe, Tomoshi Tsuchiya

**Affiliations:** 1 Department of Thoracic Surgery, University of Toyama, Toyama, Toyama, Japan; 2 Division of Thoracic Surgery, Kurobe City Hospital, Kurobe, Toyama, Japan; 3 Department of Chest Surgery, St. Marianna University School of Medicine, Kawasaki, Kanagawa, Japan

**Keywords:** staple line granuloma, tranilast, thoracoscopic surgery

## Abstract

**INTRODUCTION:**

A staple line granuloma (SG) in the lung, which arises adjacent to a staple line after lung surgery, is often difficult to differentiate from a stump recurrence. We report two cases of lung tumors that were suspected of being SGs, and the tumors resolved after the use of oral tranilast.

**CASE PRESENTATION:**

Case 1 is a 71-year-old woman who underwent a right S8 segmentectomy for lung adenocarcinoma (pT1miN0M0, stage IA1). A follow-up chest computed tomography (CT) scan, which was performed 9 months after surgery, revealed a mass adjacent to the staple line. The lesion disappeared by the 3rd month after administration of tranilast with no recurrence. Case 2 is a 70-year-old woman who underwent wedge resection for metastatic lung cancer originating from renal cancer. A follow-up chest CT scan, which we obtained 8 months after surgery, revealed a nodule adjacent to the staple line. The lesion disappeared by the 4th month after administration of tranilast with no recurrence.

**CONCLUSIONS:**

Administration of tranilast can be a safe and effective diagnostic treatment for SG, when the treatment is performed with strict imaging follow-up and histologic biopsy in mind.

## Abbreviations

CT
: computed tomography
FDG
: fluorodeoxyglucose
PET
: positron emission tomography
SGs
: staple line granulomas
SUVmax
: maximum standardized uptake value

## INTRODUCTION

Granulomas sometimes arise adjacent to staple lines after lung surgery. They can be due to a foreign body reaction to the staples or due to a biological response to an infection by pathogens such as acid-fast bacilli that can occur in lung tissue with impaired airway clearance or crushed lung tissue near a staple line.^1–8)^ Staple line granulomas (SGs) that were resected to differentiate the tumors from stump recurrences have been reported.^1–8)^

Tranilast, which is *N*-(3,4-dimethoxycinnamoyl)-anthranilic acid (Rizaben; Kissei Pharmaceuticals, Matsumoto, Japan), is a derivative of the amino acid tryptophan.^9)^ This oral drug inhibits the production and release of various inflammatory mediators and cytokines by inflammatory cells.^9)^ Attempts to shrink granulomas given the diverse pharmacological properties of tranilast have been described.^10–13)^ We report two cases of lung tumors suspected of being SGs that arose adjacent to staple lines, and we used tranilast to resolve the tumors.

### Ethical statement

Because the use of tranilast for granulomas is off-label, this treatment was reviewed and approved by the Kurobe City Hospital Institutional Review Board. Informed consent was also obtained from the patients for the treatment.

## CASE PRESENTATION

### Case 1

**Fig. 1:**
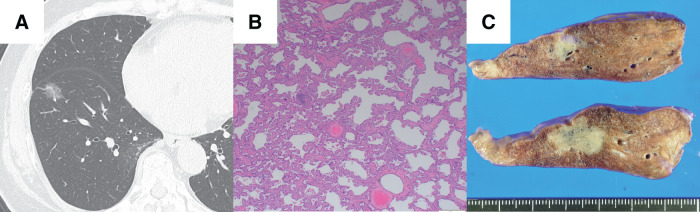
Case 1. A chest CT scan revealed a ground-glass opacity in the right lower lobe (**A**); the lesion was diagnosed after a right S8 segmentectomy as being a minimally invasive adenocarcinoma (hematoxylin and eosin staining) (**B**); an adequate surgical margin was obtained (**C**).

**Fig. 2:**
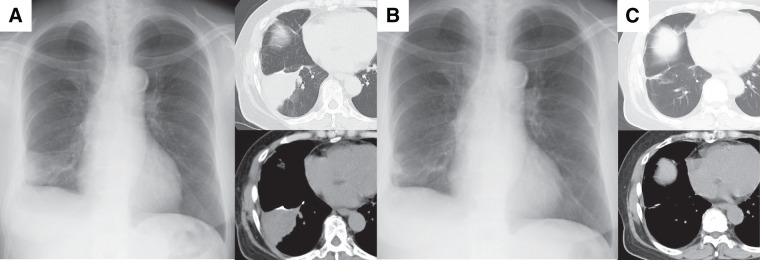
Postoperative CT images of Case 1. A mass adjacent to the staple line was found on the postoperative CT scan and chest X-ray, which were obtained 9 months after surgery (**A**). A chest X-ray, which was performed 1 month after administration of tranilast (**B**); it showed that the mass shrank quickly in size; by the 3rd month after tranilast administration the CT scan showed that the mass had disappeared (**C**).

A 71-year-old woman, who had no history of allergy or respiratory infection, underwent thoracoscopic right S8 segmentectomy after detection of a ground-glass opacity in the right lower lobe of the lung by chest CT (**Fig. 1A**). The pathological diagnosis of the tumor was minimally invasive adenocarcinoma (**Fig. 1B**), pT1miN0M0, stage IA1, and during the operation we obtained an adequate surgical margin (**Fig. 1C**). A follow-up CT scan and chest X-ray, which we obtained 9 months after surgery, revealed a mass adjacent to the staple line (**Fig. 2A**). Bronchoscopy revealed no airway stenosis that could have caused atelectasis, and a bronchoscopic biopsy revealed no malignancy. Because the staple line was located in the margins of the tumor, an SG was suspected on the basis of the CT finding, and oral tranilast (300 mg/day) treatment was started. After tranilast administration, the tumor size shrank quickly (**Fig. 2B**), and the lesion disappeared by the 3rd month after administration of tranilast (**Fig. 2C**). Tranilast was discontinued after confirmation of the disappearance of the tumor on chest CT. Four years have passed since cessation of tranilast treatment, and no relapse has occurred.

### Case 2

**Fig. 3:**
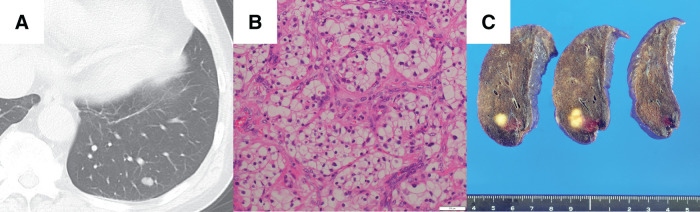
Case 2. A chest CT scan revealed a nodule in the left lower lobe of the lung (**A**); the lesion was diagnosed after wedge resection as being a renal cancer metastasis (hematoxylin and eosin staining) (**B**); an adequate surgical margin was obtained **(C)**.

**Fig. 4:**
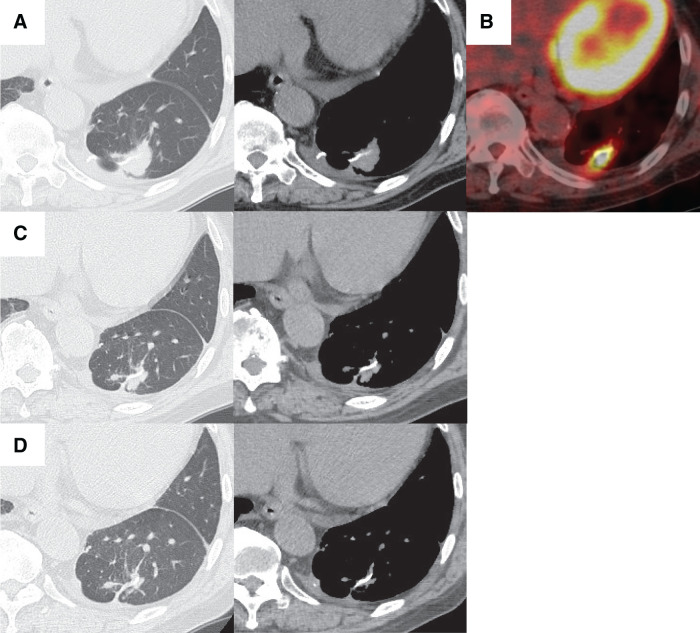
Postoperative CT images of Case 2. A nodule adjacent to the staple line was found on the postoperative CT scan, which was obtained 8 months after surgery (**A**). The SUVmax of FDG uptake on PET/CT was 10.3 (**B**). The nodule shrank in size on the CT scan, which was performed 1 month after administration of tranilast (**C**), and the nodule had disappeared by the 4th month after tranilast administration (**D**). FDG, fluorodeoxyglucose; PET, positron emission tomography; SUVmax, maximum standardized uptake value

A 70-year-old man, who had no history of allergy or respiratory infection, underwent CT 5 years after surgery for renal cancer, and a nodule was found in the left lower lobe of the lung (**Fig. 3A**). Metastatic lung cancer was suspected, and a thoracoscopic wedge resection was performed. The pathological diagnosis of the tumor was renal cancer metastasis (**Fig. 3B**), and during the operation we obtained an adequate surgical margin (**Fig. 3C**). A follow-up CT scan, which we obtained 8 months after surgery, revealed a nodule adjacent to the staple line (**Fig. 4A**). Positron emission tomography (PET)/CT studies revealed FDG uptake in the nodule, with a SUVmax of 10.3 (**Fig. 4B**). Because the staple line was located in the margins of the tumor, an SG was suspected on the basis of the CT finding, and oral tranilast (300 mg/day) was started. The tumor size shrank quickly (**Fig. 4C**) as a result of tranilast administration, and the lesion disappeared by the 4th month after administration of tranilast (**Fig. 4D**). Tranilast was discontinued after confirmation of the disappearance of the tumor on chest CT. Three years and 6 months have passed since cessation of tranilast treatment, and no relapse has developed.

## DISCUSSION

Although stump recurrences should be the first type of tumor suspected for tumors arising from staple lines after lung cancer surgery, SGs can also be considered in the differential diagnosis, because granulomas can occur adjacent to staple lines after lung surgery.^1–8)^ Granulomas are tumors that form as a defensive response to suppress foreign or infectious agents that cannot be eliminated by individual phagocytes.^14)^ One factor that may cause an SG is a foreign body reaction to staples that are used during surgery.^1)^ However, titanium, which is the material utilized to make staples, rarely causes a metal allergy because of its high biocompatibility. Also, SGs tend to arise in lung tissues adjacent to the staple line in cases of segmentectomy or wedge resection, and these procedures are more likely to be non-anatomic resections compared with lobectomy or have a risk of crushed lung tissue in stapling under excessive tension used to secure the surgical margin; in addition, acid-fast bacilli are often detected by microscopy or culture examination of resected specimens.^2,6)^ For these reasons, SGs are likely to form as a biological response to infection by pathogens such as acid-fast bacilli that can occur in lung tissue with impaired airway clearance or crushed lung tissue near the staple line. In recent years, many studies have reported the efficacy of sublobar resection for early stage lung cancer,^15,16)^ and its application has been increasing. The number of cases of SGs requiring differentiation from stump recurrence may therefore also increase. The following CT image features have been reported as useful findings in differentiating SGs from stump recurrences.^6–8)^ In cases of SGs, tumors tend to grow from staple lines as their base lines, so in many cases staple lines are at the margins of the tumors. However, staple lines tend to be surrounded by tumors in cases of stump recurrences. In the two cases reported here, the staple lines were located at the tumor margins on the CT images, which provided evidence to suggest the presence of SGs as in previous reports. However, these features are only image findings, so that establishing additional diagnostic treatment for differentiating SGs from stump recurrences is needed.

Tranilast is used to treat type I hypersensitivity disorders such as bronchial asthma and hypertrophic scars because it can inhibit histamine release from mast cells and collagen synthesis by fibroblasts. It is also utilized in various immunological diseases because of its diverse pharmacological effects.^9)^ During granuloma formation, large numbers of monocytes and macrophages in the peripheral blood gather at the lesion, and cytokines produced by helper T cells, especially interferon-γ (IFN-γ), play an important role in this process.^14,17,18)^ Aggregated macrophages differentiate into multinucleated giant cells or epithelial cells by means of cytokines such as tumor necrosis factor-α (TNF-α), interleukin (IL)-4, and IL-13, which are produced by helper T cells or macrophages themselves, after which these cells surround foreign bodies or infectious agents to form granulomas.^14,17)^ In addition, fibroblasts that proliferate at the margins of granulomas synthesize collagen and promote fibrosis to suppress lesion expansion, and transforming growth factor-β (TGF-β) and various ILs have important roles in this process.^14)^ Tranilast can suppress the production of a wide variety of cytokines including IFN-γ, TNF-α, TGF-β, and various ILs. These mechanisms may be related to the shrinking of SGs as an effect of tranilast.

Although a tissue biopsy should be performed as quickly as possible for lung tumors with a high probability of stump recurrence, performing a bronchoscopic or CT-guided biopsy is sometimes difficult because of the anatomic location of the tumor. Surgery is often indicated as diagnostic treatment for such undiagnosed tumors, although reoperation of the ipsilateral residual lung, which often has severe intrapleural adhesions, is a high-risk procedure for patients. Tranilast also has few side effects.^9)^ Thus, we expect that tranilast can be applied as a safe and effective diagnostic treatment for lung tumors suspected of being SGs that are not likely to be stump recurrences. Such tumors include those with sufficient surgical margins at the initial surgery and also with characteristic CT findings, which are suspected of being SGs, provided the treatment is performed with plans for strict imaging follow-up and histologic biopsy.

## CONCLUSIONS

Although stump recurrences should be initially suspected for tumors arising from staple lines after lung cancer surgery, SGs can also be considered in the differential diagnosis. For lung tumors suspected of being SGs with sufficient surgical margins at the initial surgery and also have characteristic CT findings of SGs, administration of tranilast can be an effective diagnostic treatment, provided the treatment is performed with plans for strict imaging follow-up and histologic biopsy.

## ACKNOWLEDGMENTS

We would like to thank Judith B. Gandy (Precision Editing) for the English language review.

## DECLARATIONS

### Funding

None declared.

### Authors’ contributions

Article writing: AU, YS.

Data collection: AU, YS.

Clinical practice: AU, YS, TH, KT.

Proofing: YS, TT.

All authors take responsibility for all aspects of the study.

All authors read and approved the manuscript.

### Availability of data and materials

The data will be shared on reasonable request to the corresponding author.

### Ethics approval and consent to participate

This study was approved by the Kurobe City Hospital Institutional Review Board (No. 302).

### Consent for publication

Written consent was obtained from the patients for publication.

### Competing interests

The authors declare that they have no competing interests.
